# Biocatalysts Immobilized in Ultrathin Ordered Films

**DOI:** 10.3390/s101110298

**Published:** 2010-11-16

**Authors:** Jadwiga Sołoducho, Joanna Cabaj

**Affiliations:** Department of Medicinal Chemistry and Microbiology, Faculty of Chemistry, Wroclaw University of Technology, Wybrzeze Wyspianskiego 27, 50-370 Wroclaw, Poland; E-Mail: joanna.cabaj@pwr.wroc.pl

**Keywords:** biosensors, enzyme immobilization, Langmuir-Blodgett technique, enzyme activity, AFM

## Abstract

The immobilization of enzymes and other proteins into ordered thin materials has attracted considerable attention over the past few years. This research has demonstrated that biomolecules immobilized in different [Langmuir-Blodgett (LB)/Langmuir-Schaefer (LS)] matrixes retain their functional characteristics to a large extent. These new materials are of interest for applications as biosensors and biocatalysts. We review the growing field of oxidases immobilized onto ordered Langmiur-Blodgett and Langmuir-Schaefer films. Strategies for the preparation of solid supports and the essential properties of the resulting materials with respect to the envisaged applications are presented. Basic effects of the nature of the adsorption and various aspects of the application of these materials as biosensors, biocatalysts are discussed. Outlook of potential applications and further challenges are also provided.

## 1. Introduction

Biosensors generally offer simplified reagentless analyses for a range of biomedical and industrial applications and for this reason this area has continued to develop into an ever-expanding and multidisciplinary field during the last years. For any sensor, fast response and reversibility are often paramount. In any solid-state sensor, the analyte molecules need to diffuse into and react with the acting sensing component and every reaction product must diffuse out. Therefore, the thinner the sensing layer is, the shorter time this will take and then speed and reversibility of sensor may be increased.

While many biocatalysts can be used as free enzymes or as whole cells, immobilization of biocatalysts provides additional features that can significantly improve biocatalytical reactions. The technology required for immobilization of biological catalysts has expanded greatly for the last 30 years. A consequence of the explosion of the technology is now a bewildering array of permutation for options for the immobilization of biological materials.

Much research has been dedicated over many years into the fabrication of thin films of wide variety of materials, often just one molecule thick [1]. A wide variety of techniques can be used for fabrication this type of films, *i.e.*, Langmuir-Blodgett (LB) and Langmuir-Schaefer (LS) techniques or by using self-assembly monolayers.

LB film technology is one of the important technologies for future molecular electronic and bioelectronic devices. Protein-incorporating LB films were extensively studied from the standpoint of application for bioelectronic devices. The LB technique is therefore ideal for creating well-ordered enzyme systems which may be used in biosensing. Fabricating ordered films involves formation of stable monolayers at the air-liquid interface to be subsequently transferred onto a solid support, which then requires a detailed investigation on the mechanisms through which different materials interact at the interface. This is especially important when biomolecules are to be immobilized in LB films, whose biological activity strongly depend on the molecular arrangements [2].

Some unconventional materials form a Langmuir film so rigid that it does not produce high quality deposited films when using the LB technique. As material is removed from the monolayer during the first deposition, there may be very little molecular reorganization on the subphase surface. This results in poor transfer of material on subsequent LB depositions in the same region. A solution to this problem is to deposit using the LS deposition technique. By positioning the substrate horizontally, there is no requirement for the monolayer material to reorganize, as a large area of film is deposited instantly when the horizontal substrate touches the monolayer. This approach will yield a greater number of layers from each compressed film but makes the assumption that each layer was deposited at the correct surface pressure, as measured far across the film at the surface pressure sensor. A large advantage of the LS deposition technique is the ability to deposit a single monolayer during each excursion, in comparison to the deposition of two layers when using the LB technique. The technique is very useful for deposition of proteins and rigid layers [3].

The adsorption of proteins from solution onto solid surfaces has attracted much attention due to its scientific importance and applications in many areas [4]. The adsorption (immobilization) of proteins on inorganic materials is crucial because of the potential to improve the stability of enzymes under extreme conditions. The controlled adsorption of proteins is essential in the fields of enzymatic catalysis, biosensors, and disease diagnostics.

Very few enzymes or proteins can form sole LB films, but most protein molecules can be incorporated to a solid surface by adsorption from solutions and their subsequent binding to an aliphatic acid film [1] *via*. a -COOH group or by covalent cross-linking (including in LB film). In some cases, immobilization of proteins on the solid substrate can improve the sensing stability and allow for their reuse (Table 1).

Herein, we discuss biological sensors based on enzymes (oxidases) adsorbed on ultrathin layers built of lipid-like structures and conducting compounds. Conducting structures formed by deposition are of great interest as sensors. Suitable polymers can be incorporated into LB structures and for example, polyaniline/glucose oxidase LB film can be deposited and used as an electrochemical sensor for glucose with a linear response to 30 mM [12]. Other conducting polymers retain their electroactivity and detect glucose or urea [13,14].

Among enzymes, laccases and tyrosinases are two groups of phenol oxidases that catalyze the transformation of a large number of phenolic and non-phenolic aromatic compounds. Abundant information is available in the literature on the use of free and immobilized phenol oxidases in several application areas [15,16]. To date, however, an exhaustive overview in the basic aspects of immoblilization of laccase and tyrosinase has been lacking. To retain the enzymes’ specific biological function, their immobilization on a solid matrix is a key factor in preparing biosensors. So far several immobilization strategies have been commonly used to immobilize small molecules onto appropriately functionalized glass slides, including covalent immobilization with Staudingeer ligation [17]. Immobilization methods for tyrosinase such as physical adsorption, covalent cross-linking, incorporation within carbon paste, immobilization in polymer films, entrapment in cyro-hydrogel and some sol–gel matrices have also been reported in the literature [10].

## 2. Results and Discussion

### 2.1. Enzymatic Sensor Devices

**Figure 1.**
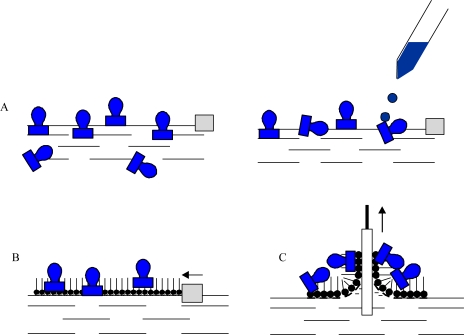
Three approaches to form enzyme mononlayers through Langmuir and LB technique; 
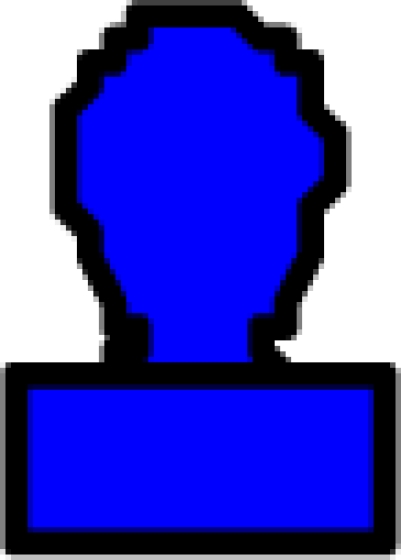
 - protein (*i.e.*, enzyme), 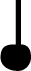 - amphiphilic molecule (*i.e.*, phospholipids).

Enzymes are intrinsically amphiphilic molecules containing both hydrophobic and hydrophilic moieties since they are aminoacids. Therefore, proteins are readily adsorbed to various interfaces (air-water, liquid-liquid, liquid-solid). The adsorption of proteins on a water surface can be accomplished by using two different ways. The first method is injection of proteins into water’s subphase and monitoring the dynamic adsorption processes of proteins at the air-water interface. The second approach is to spread the solution of proteins at the air-water interface, and then protein molecules at the interface are compressed to form a Gibbs monolayer [Figure 1(A)]. Due to the intrinsic amphiphilic properties of proteins, many enzymes readily form a film that is similar to a Langmuir monolayer if the desorption from the interface to the aqueous subphase were kinetically slow or subjected at slow adsorption at water surface.

**Figure 2.**
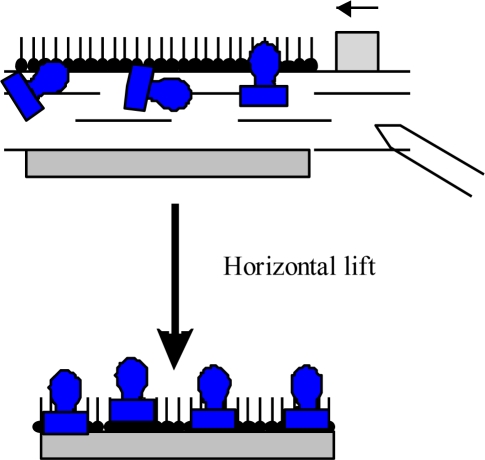
Langmuir-Schaefer deposition of Langmuir monolayer onto the solid support; 
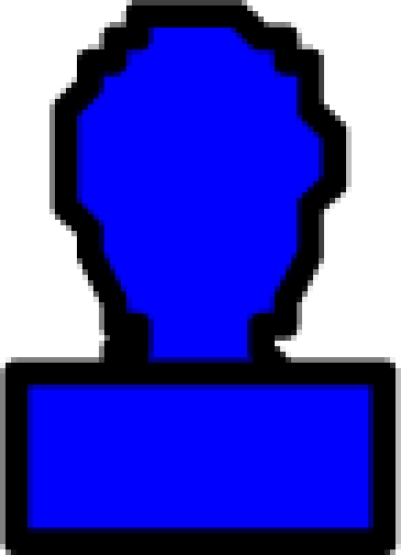
–protein (*i.e.*, enzyme), 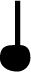
–amphiphilic molecule (*i.e.*, phospholipids).

Other than adsorption onto a pure water surface, proteins also readily adsorb on an existing lipid monolayer formed at an air-water interface. In this situation, enzymes can be inserted into the lipid monolayer or attached only to the polar head-groups of the lipids [Figure 1(B)]. The Langmuir monolayer of a traditional lipid is easily prepared and can be controlled. Surface pressure and composition of lipid layer greatly affect the protein adsorption and, therefore the monolayer structure [18]. Direct adsorption of proteins onto a LB film of lipid is also a commonly used technique to prepare enzyme monolayers [Figure 1(C)] [9]. Then, lipids are deposited from the air-water interface to a solid support to form one- or multiple-layered LB film. After the formation of one protein layer, multilayers can be prepared by alternately depositing one layer of lipid followed by protein adsorption. Both the Langmuir-Blodgett and Langmuir-Schaefer (Figure 2) techniques have been reported for the deposition of proteins on the solid support.

However, it was observed that the horizontal lift LS method is better suited to proteins and provides homogenous and reproducible coverage of the substrate [3]. In both cases the protein is attached to the support by non-covalent bonding. The formation of an ordered film on the water surface is associated with the amphiphilic properties of deposited molecules. A typical example of an amphiphilic molecule is a fatty-acid molecule consisting of a hydrophilic polar head and a hydrophobic hydrocarbon chain as well as lipids, then these structures are very often used as a mixing agents in preparation of protein layers.

The first attempt to obtain a protein film on the water surface was made by Langmuir and Schaefer [19]. The ability of water-soluble proteins to form films was first explained by the unfolding of polypeptide chains on the water surface and opening of hydrophobic groups. But later on it was established that a number of water-soluble proteins forming a monolayer preserve their functional activity [9]. It is known, at present, that LB/LS films can be produced from a wide class of proteins. In case it is necessary to obtain an LB/LS film from proteins unstable at interfaces, these proteins can be either attached to the charged heads of a lipid layer formed on the water surface [20] or built into layers forming the protein-lipid model membrane sequence. For instance, according to Girart-Ergot *et al.* [2] enzyme bioactivity in mixed lipid LB films is preserved due to the lipid molecular assembly protects the enzyme, positioning the polypeptide moiety in such a way as to allow the recognition and signal events. In fact, phospholipids have been used as protecting agents for several types of material, not only for membrane cell proteins [21] but also for polysaccharides [22] or synthetic polymers [23].

**Figure 3.**
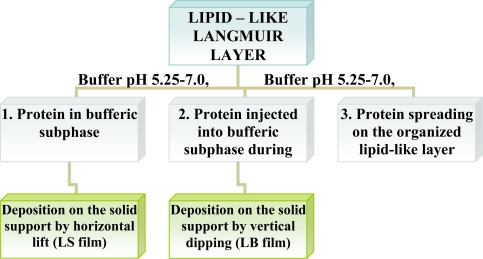
Approaches to form protein LB/LS films.

Dipalmitoylphosphatidylglycerol (DPPG) is extensively applied in studies involving mimetic systems in the form of Langmuir monolayers [24], LB films [11,25]. Phospholipids are insoluble molecules, so their evaporation and dissolution is neglible. The major cause for the poor respreading ability of phospholipids monolayers is film “collapse”. This collapse phenomenon was first described by Langmuir to explain the compression-extension hysteresis in surface pressure-area curves [26]. Since then, the collapse mechanism of an insoluble monolayer, such as long-chain fatty acids with chain lengths of more than eighteen carbons, has been studied [27]. Conditions for the preparation and transfer of the LB/LS films and compositions of the films are listed in Figure 3 and Table 2.

**Figure 4.**
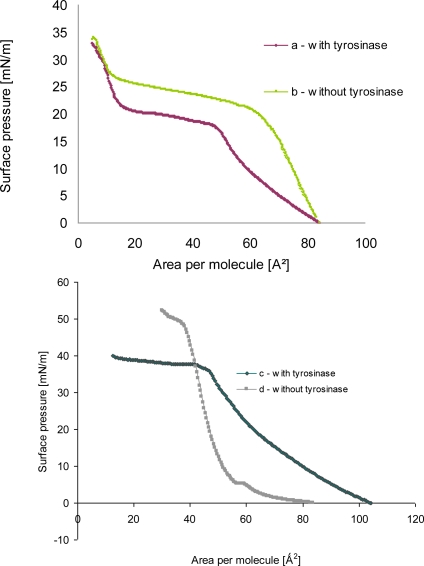
Surface pressure—area isotherms of tyrosinase incorporated in linoleic acid (LA) –**a**, or phospholipids −**c** layer and protein free film **b, d** at 295 K on HEPES buffer (pH 6.52); 4a [11].

A monolayer of phenoloxidase molecules at the air-water interface was expected to be formed by fatty acid or phospholipids layers having negative charge in buffer solution because of charge of proteins. The formation of tyrosinase—phospholipids as well as tyrosinase—linoleic acid hetero monolayer at the air-water interface was experimentally proven from the π-A isotherms as shown in Figure 4.

The π-A isotherm of amphiphile monolayer after proteins adsorption was shifted to the right direction in comparison with that of amphiphile mixture before proteins adsorption (a, b in Figure 4). It was guessed that the shift of π-A isotherm of mixed amphiphiles layer after enzymes adsorption would result from the increase of area occupying per molecule at the air-water interface.

The isotherm of monolayer **a** (Figure 4) after tyrosinase adsorption showed that the surface pressure gradually increased until 27 mN/m, also during compression the slope of curve firmly decreased. The slope was turned upward more steeply as the area was approached to the condensed monolayer region. It might be due to the fact either that the liquid—expanded films were organized into two-dimensional or that the hindered molecular orientation occurred, which resulted in the discontinuity of compressibility [11].

In the surface pressure-area isotherm of pure phospholipid monolayer b (in Figure 4) plateau was found that represents the transition from the liquid-expanded phase to liquid-condensed phase. Upon introducing tyrosinase in the suphase, the isotherm became more expanded. The increase in area may be due to the adsorption of the enzyme at the monolayer. Moreover, the liquid-condensed (LE-LC) transition was not distinguishable for the mixed monolayer, *i.e.*, a plateau was not defined.

When tyrosinase was injected into the subphase of spread lipid monolayer, an increase in pressure was observed, whose amplitude depended on the monolayer initial surface pressure. This indicated a large affinity of protein toward phospholipid, probably driven by electrostatic interactions as well as interpenetration of the polypeptide moiety into the alkyl chains of the lipid, which characterize hydrophobic interactions. Other possibilities also exist, such as changes due to cooperative interaction between phospholipids and tyrosinase molecules, which may facilitate or hamper the packing. It is also possible that tyrosinase molecules may adsorb below the polar heads of phospholipids, forming a kind of “subsurface”.

### 2.2. Detection of Biosensing Effect of Fabricated LB/LS Protein Films

Since the immobilization of laccase on LB films was achieved through the cross-linking reaction of with glutaraldehyde [28], its amount reflects the immobilized enzyme activity. Another important method of enzyme immobilization is the process of adsorption. Adsorption of an enzyme onto a solid matrix is probably the simplest and fastest way to prepare immobilized enzymes. The method relies on a non specific physical interaction – based on weak forces, such as van der Waals or dispersion forces—between the enzyme and the surface of the matrix which is brought about by mixing a concentrated solution of enzyme with the solid. The active site is normally unaffected and nearly full activity is observed.

**Figure 5.**
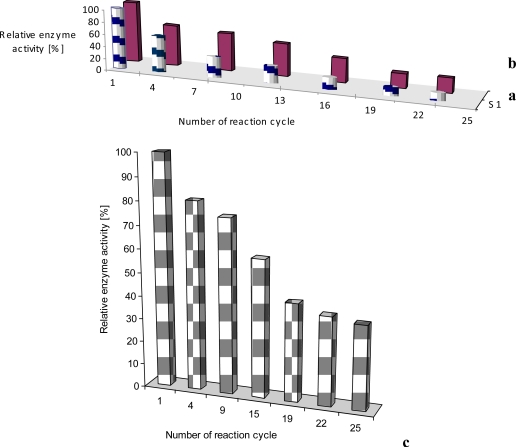
Immobilized laccase–**a**, tyrosinase–**b**, [11] and glucose oxidase–**c** activity during repeated reaction cycle in presence of different reagents (ABTS *tert*-buthylcatechol, glucose) as substrate.

In our case, laccase incorporated into obtained film had an initial enzyme activity of merely 70% of the activity of the native laccase. In the case of tyrosinase initial protein activity was close to about 4% of that of free protein [11]. As seen from Figure 5, the sensing activity of the phenoloxidases incorporated into LB films is rather stable and reproducible, especially for the laccase sensor. The sensing activity of the laccase, adsorbed onto LB films was reproducible during up to 25 incubation cycles (repeated reaction of oxidizing reagent catalyzed by immobilized enzyme). The observed decrease of enzyme activity is rather small and the adsorbed protein is active for a few months (stored at 4 °C). Very similar situation was found in case of glucose oxidase incorporated in phospholipids LS layer (Figure 5c). The sensing activity of the oxidase, adsorbed onto LS film was reproducible during up to 25 incubation cycles (repeated reaction of oxidizing reagent catalyzed by immobilized enzyme).

ABTS as a standard enzyme activity indicator used for the reaction catalyzed by laccase showed much higher protein activity compared to the natural reagents like *o*-aminophenol or catechol. In the case of natural reagents (Table 3) the laccase activity in the film, although is as low as 68–1% of the activity of laccase in presence of 2,2′-azino-bis(3-ethylbenzthiazoline-6-sulphonic acid)-ABTS, is stable and repeatable [11].

### 2.3. Conjugated Electrical Connectors

Understanding the role and mechanism of action of these mediators is a practical issue. For example, ABTS is the most common mediator for laccase activity but not the most efficient one. ABTS has been also described as an efficient mediator of laccase activity towards non-phenolic substrates [29]. Mediation by ABTS is suggested to involve the oxidation of the substrate by an electron transfer step [30]. In this event, the reactivity of mediation correlate with the oxidation potentials within a series of structurally homogeneous substrates, the easier to oxidase substrate owing to react more easily [31].

**Figure 6.**
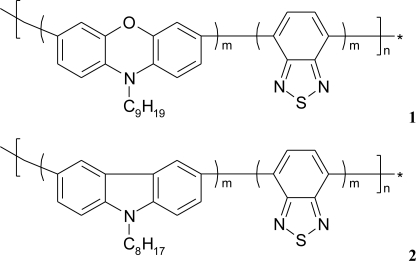
Conjugated polymers—poly[(*N*-nonylphenoxazine-3,7-diyl-*alt*-(1,2,3-benzothiadiazole)]—**1**, and poly[(*N*-octylcarbazole-3,6-diyl-*alt*-(1,2,3-benzothiadiazole)]—**2**.

**Figure 7.**
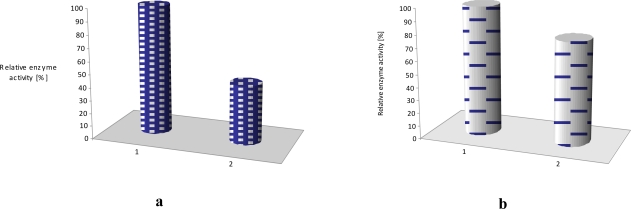
Activity of laccase–**a** [11] and glucose oxidase–**b** immobilized in LB/LS film, 1- with polymer as mediator (laccase–polymer **1**, glucose oxidase–polymer **2**), 2- without a polymer mediator.

If additional poly[(*N*-nonylphenoxazine-3,7-diyl-*alt*-(1,2,3-benzothiadiazole)] (**1**) as well as poly-[(*N*-octylcarbazole-3,6-diyl-*alt*-(1,2,3-benzothiadiazole)] (**2**) molecules (Figure 6), acting as an electron mediator, are present in the system they significantly enhance a reagent’s mediating efficiency, as displayed in Figure 7. The presence of additional molecules with conjugated bonds (**1**, **2**) in the system improves ABTS mediating efficiency significantly. If the additional benzothiadiazole—based copolymers **1** or **2** molecules, acting as an electron mediator are present in the system they significantly enhance the mediating efficiency of reagents.

The effect of equimolar addition of **1** into matrix of linoleic acid and octadecyltrimetyl-ammonium bromide is depicted in Figure 7; an enzyme activity increased more than two times in case of laccase and it retained ca. 70% of its initial activity for as long as 4 months (stored in the buffer, at 4°C) [11]. Similar situation was observed in case of LS film of glucose oxidase (GOx) incorporated in phospholipids layer. The polymer **2** has affected the enzyme activity, it was 20% higher than activity of GOx immbilized without conjugated compound [Figure 7(b)].

The surface of every molecule in the mixed LB/LS film (calculated from area per molecule) suggests that benzothiadiazole-based copolymer molecules **1** and **2** are squeezed from the carboxylic heads of linoleic acid or phospholipid layer with their aliphatic chains being parallel. This “multilysinic” bonding of enzymes with the LB/LS films opens in some respects the access to its active centres. Moreover, the grafting of enzyme into LB/LS layers can provide better electrosterical stabilisation, due to the high molecular weight of the protein [32,33].

### 2.4. AFM Study of Ultrathin Films

**Figure 8.**
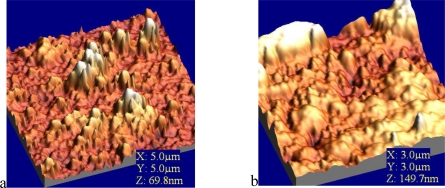
AFM images of linoleic acid/octadecyltrimethylammonium bromide/**1**/laccase–**a** and linoleic acid/octadecyltrimethylammonium bromide **/1**/tyrosinase LB film–**b**. All images are 3 μm × 3 μm [11].

In order to visualize the deposition of phenolooxidase (laccase, tyrosinase) thin films and to analyze the surface density of prepared protein films, atomic force microscopy (AFM) was employed. The enzyme molecules were fairly well deposited onto solid substrate. Immobilized phenoloxidases were observed as an aggregated pattern in solid-like state with keeping their characteristic random cloud-like structure (Figure 8).

These obtained values were attributed to the process of immobilization of comparatively large molecule aggregates of enzyme (laccase, tyrosinase) incorporated into LB film. This leads to the conclusion that an agglomerate of enzymes is formed rather than an organized monolayer at the air/aqueous interface. The AFM results showed that the effect could be also associated with changes in the enzyme conformations. A monolayer rearrangement, such as two-dimensional formation or hindered molecular orientation, might take place during the phase transition behaviour resulted in the molecular aggregates on the protein layer [11].

**Figure 9.**
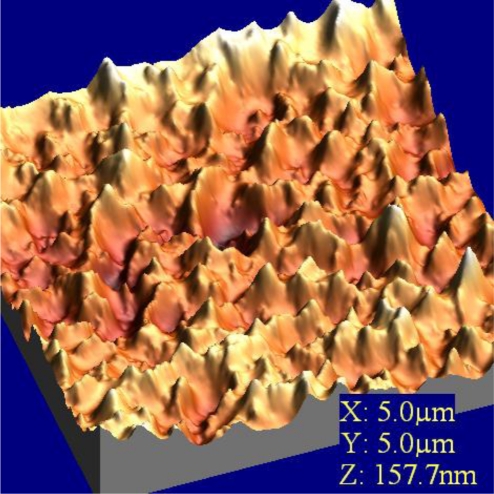
Surface topography of phospholipids/**2**/GOx LS film.

Immobilization of glucose oxidase onto the activated mica surface revealed sometimes big irregularities with 100–400 nm diameter and 50 nm height on the surface [36]. This means that during the immobilization, enzymes form huge conglomerates, as in natural conditions the dimeric molecule of glucose oxidase has dimensions even 5 × 8 nm [37]. The decrease of the concentration of the silanizing agent to 0.1% with no changes in other immobilization steps, led to the disappearance of the big conglomerates and showed homogeneous filling of the surface with the enzyme. AFM images of glucose oxidase-incorporating arachidic acid LB films showing clear surface morphology observed over a 100–600 nm area. This surface is stable and is unchanged after several minutes of continuous observation [38]. Very similar situation was observed in case of LS film of glucose oxidase adsorbed to phospholipids (Figure 9). The layer is homogenous with clear morphology and roughness found as 1.8 nm.

### 2.5. Oxygen Dependence

Since oxidase-based devices rely on the use of oxygen as the physiological electron acceptor, they are subject to errors resulting from fluctuations in oxygen tension and the stoichiometric limitation of oxygen [39]. These errors include changes in sensor response and a reduced upper linearity limit.

Several avenues have been proposed for addressing this oxygen limitation. One approach relies on the use of mass transport-limiting films (such as polyurethane or polycarbonate) for tailoring the flux of glucose and oxygen, *i.e.*, increasing the oxygen/glucose permeability ratio [40–42]. A two dimensional cylindrical electrode, designed by Gough’s group [41,42], has been particularly attractive for addressing the oxygen deficit by allowing oxygen to diffuse into the enzyme region of the sensor from both directions while glucose diffuses only from one direction (of the exposed end). This was accomplished by using a two-dimensional sensor design with a cylindrical gel containing GOx and an outside silicone rubber tube which is impermeable to glucose but highly permeable to oxygen.

The oxygen limitation of glucose biosensors is addressed by designing oxygen-rich enzyme electrodes [43,44]. The biosensor based on a LS film in which GOx is incorporated into phospholipids film could be an opportunity for material allowing the oxygen diffuse. The internal flux of oxygen can thus support the enzymatic reaction, even in oxygen-free glucose solutions. It is also possible to circumvent the oxygen demand issue by replacing the GOx with glucose dehydrogenase (GDH), which does not require an oxygen cofactor [45].

### 2.6. Modified Enzyme Electrodes

Chemical modification of GOx with electron-relay groups also represents another attractive route for facilitating the electron transfer between the GOx redox center and the electrode surface. Hill described the covalent attachment of ferrocene monocarboxylic acid to the lysine residues of GOx using isobutyl choloformate [46], whereas Degani and Heller [47] used carbodimide coupling for attaching the same mediator to GOx. Such covalent attachment of ferrocene groups led to direct oxidation of the flavin center of GOx. Furthermore, the carbodimide-based covalent attachment to tetrathiafulvalene to the peptide backbone of GOx has been described [48].

**Figure 10.**
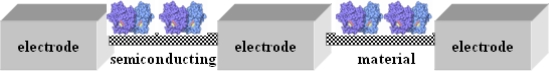
Modified GOx electrode

Glucose biosensors with extremely efficient electrical communication with the electrode can be generated by the enzyme reconstitution process. Riklin *et al.* [49] reported on an elegant approach for modifying GOx with electron relays and obtaining efficient electrical contact. In our case we modified the electrode by biocatalytical active LS layer build of phospholipids and GOx (Figure 10).

### 2.7. Requirements

The major requirements of clinically accurate *in-vivo* sensors have been discussed in several review articles [39,40,50–52]. The ideal sensor would be one that provides reliable real-time continuous monitoring of different species in medical as well environmental diagnostics throughout the day with high selectivity and speed over extended periods under harsh conditions. The challenges for meeting these demands include rejection of the sensor by the body, miniaturization, long-term stability of the enzyme and transducer, oxygen deficit, *in-vivo* calibration, short stabilization times, baseline drift, safety, and convenience. The sensor must be of a very tiny size and proper shape that allows for easy implantation to cause minimal discomfort. Undesirable interactions between the surface of the implanted device and biological medium cause deterioration of the sensor performance upon implantation and proved to be the major barrier to the development of reliable *in-vivo i.e.*, glucose probes.

## 3. Conclusions

For nearly 50 years we have witnessed tremendous progress in the development of chemical and biological sensors. Elegant research on new sensing concepts, coupled with numerous technological innovations, has thus opened the perspective to applications of sensors and biosensors.

Using modified or unmodified conducting structures as a receptor material or as one of components of the receptor layer in chemical sensors offer a wide range of applications as one of the most stable detection layers. Furthermore, enzymes immobilized in thin films constitute nearly 85% of the world market for biosensors. Major fundamental and technological advances have been made for also enhancing the capabilities and improving the reliability of *i.e.*, phenol or glucose measuring devices.

A heterogeneous LB/LS film, consisting of amphiphilic arylenes and long-chain lipids provides one of successful way for catalyst immobilization. Enzymes immobilized by this could be alternative method for routine analysis.

As this field enters its fifth decade of intense research, significant efforts that couple the fundamental sciences with technological advances are expected. This stretching of the ingenuity of researchers will result in advances, including the use of nanomaterials for improved electrical contact between the redox centre and electrode supports.

## Figures and Tables

**Table 1.** Comparison of selected protein sensors immobilized in thin films.

***Immobilization method***	***Sample thickness***	***Response time***	***Stability***	***Reference***
Glucose oxidase (GOx) crosslinked with glutaraldehyde on Pt electrode	Not reported	0.4 min	10 days	[5]
Glucose oxidase (GOx) electrodeposited with polyaniline	1–12 nm of polymer	2–3 min	Not reported	[6]
Catalase in phospholipid LB film	3 layer	10 min	Not reported	[7]
Lipase LS film on silanized support	1,3,5 layers	Not reported	Not reported	[8]
Horseradish peroxidase LB deposition with phospholipids	1 layer	Not reported	>2 weeks	[9]
Tyrosinase LB deposition with *N*-nonylbis(thiophene)diphenylamine and stearic acid cross-linked with glutaraldehyde	5 layers	2 min	>3 month	[10]

Laccase LB deposition with benzothiadiazole-based copolymer	5 layers	21 min	>3 month	[11]

**Table 2.** Compositions of LB/LS films and their transfer conditions.

	***Enzyme***	***Linoleic acid***	***Dipalmitoyl-phosphatidyl-glycerol***	***Octadecyl-trimetyl-ammonium bromide***	***Π (mN m^−1^)***	***Transfer.temp (K)***	***Type of deposition***
**1.**	Laccase [10]	+		+	15	295	LB
**2.**	Tyrosinase - **a** [10] – Figure 4	+		+	17	295	LB
**3.**	Tyrosinase - **c** [data not published] Figure 4		+		25	295	LS
**4.**	Glucose oxidase		+	+	27	295	LS

**Table 3.** Immobilized proteins activity in presence of various phenolic compounds [11].

***Enzyme***	***Reagent***	***Relative enzyme activity [%]***
Laccase	ABTS, 0.228mM	100
Laccase	*o*-Aminophenol, 10 mM	68
Laccase	Phenol, 10 mM	1
Tyrosinase	*tert*-Butylcatechol, 5 mM	100
Tyrosinase	l-Tyrosine, 5 mM	36

Tyrosinase	*o*-Aminophenol, 10 mM	5
